# Haemoglobin and anaemia in the SMART study

**DOI:** 10.1186/1758-2652-13-S4-P144

**Published:** 2010-11-08

**Authors:** A Mocroft, AR Lifson, G Touloumi, J Neuhaus, Z Fox, A Palfreeman, M Vjecha, S Hodder, S De Wit, JD Lundgren, AN Phillips

**Affiliations:** 1 University College London Medical School, Royal Free Campus, London, UK; 2 University of Minnesota, Minnesota, USA; 3 Athens University Medical School, Athens, Greece; 4 Medical Research Council Clinical Trials Unit, London, UK; 5 Veterans Affair Medical Centre, Washington, USA; 6 New Jersey Medical School, New Jersey, USA; 7 t Pierre Hospital, Brussels, Belgium; 8 Copenhagen HIV Program, University of Copenhagen, Copenhagen, Denmark

## Purpose of study

Data from randomized trials on the development of anaemia after interruption of therapy is not well described. We aimed to describe the development of anaemia after interruption of cART and the relationship between the development of anaemia and clinical events (AIDS, deaths or non-AIDS events) in the Strategic Management of Antiretroviral Therapy (SMART) randomised trial.

## Methods

2248 patients from the SMART study were included. We used Cox proportional hazards models to investigate development of new (<12 mg/dl for females, <14 mg/dl for males) or worsening (<8 mg/dl if anaemic at randomization) anaemia and poisson regression analyses to explore the relationship between anaemia and the development of AIDS, death or non-AIDS events.

## Results

**Figure 1:**
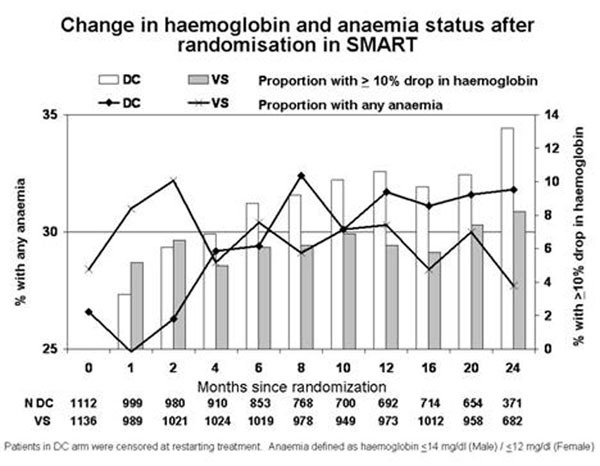


The change in haemoglobin and anaemia after randomisation to SMART is shown in Figure 1.

759 patients developed new or worsening anaemia; 420/1106 (38.0%) in the drug conservation (DC) arm and 339/1127 (30.1%) in the virological suppression (VS) arm; p<0.0001. In the first 4 months following randomization, there was no difference in the risk of new or worsening anaemia when comparing the DC arm to the VS arm (adjusted relative hazard [RH] 1.02, 95% CI 0.82-1.25, p=0.88). After the initial 4 months, patients in the DC arm had a significantly increased risk of new or worsening anaemia (adjusted RH 1.56, 95% CI 1.28-1.89, p<0.0001). 56 patients died during 5811 person-years of follow-up (PYFU), 56 developed AIDS (5728 PYFU) and 100 developed a non-AIDS event (5664 PYFU). Currently anaemic patients had an increased incidence of AIDS (adjusted IRR 2.31; 95% CI 1.34-3.98), death (2.19; 95% CI 1.23-3.87) and non-AIDS events (2.98; 95% CI 2.014.40) compared to non-anaemic patients.

## Conclusions

Patients in SMART who interrupted cART had a higher risk of new or worsening anaemia. Patients with anaemia had a higher incidence of AIDS, non-AIDS defining events or deaths; whether this relationship is causal or a consequence of the disease is not clear but suggests that anaemia, or drop in haemoglobin, might be of use as a pre-clinical marker of disease. Further research is warranted to further understand the occurrence of anaemia, its consequences and underlying pathological mechanisms.

